# Subtalar joint kinematics defined by a rotational axis translating along the posterior talocalcaneal facet

**DOI:** 10.1038/s41598-025-17948-5

**Published:** 2025-09-01

**Authors:** Naomichi Ogihara, Yuka Matsumoto, Hiroyuki Seki, Takeo Nagura, Nobuaki Imanishi, Masahiro Jinzaki, Motoharu Oishi, Hideki Endo, Gen Suwa

**Affiliations:** 1 Department of Biological Sciences, Graduate School of Science, The University of Tokyo, 7-3-1 Hongo, Bunkyo-ku, Tokyo, 113-0033 Japan; 2 Department of Orthopedic Surgery, Tachikawa Hospital, Tachikawa, Japan; 3 Department of Clinical Biomechanics, Keio University School of Medicine, Tokyo, Japan; 4 Department of Anatomy, Keio University School of Medicine, Tokyo, Japan; 5 Department of Radiology, Keio University School of Medicine, Tokyo, Japan; 6 School of Veterinary Medicine, Azabu University, Sagamihara, Japan; 7 University Museum, The University of Tokyo, Tokyo, Japan

**Keywords:** Foot, Talus, Calcaneus, Talocalcaneal joint, Helical axis, Biomedical engineering, Musculoskeletal system

## Abstract

The subtalar joint is essential for the normal function of the human foot during bipedal walking, with its kinematics being pivotal for understanding foot biomechanics, disorders, and evolution. Traditionally, the helical axis representation has been used to assess subtalar joint movement, assuming translational motion along the rotational axis. However, recent observations challenge this assumption, revealing predominantly mediolateral translation during walking. To address this discrepancy, we propose a novel method that combines a rotational axis representation with a translational axis aligned parallel to the cylindrical axis of the subtalar joint’s posterior facet. Utilizing human cadaveric lower legs, we quantified subtalar joint motion through CT scan analysis. Comparative evaluations between the conventional helical axis representation and the newly proposed cylindrical axis-based representation revealed a closer correspondence between calcaneus movement and the cylindrical axis, emphasizing the pivotal role of posterior facet morphology in subtalar joint kinematics. This innovative approach provides a more intuitive and clinically useful depiction of subtalar joint biomechanics, potentially leading to deeper insights into fundamental biomechanics and function of the human foot, and improved clinical assessment and treatment strategies for subtalar joint-related pathologies.

## Introduction

The subtalar joint is a critical component in the normal function of the human foot during bipedal walking^1–5^. An accurate representation of subtalar joint kinematics is therefore crucial not only for advancing our basic understanding of foot biomechanics^6–10^, but also for the evaluation of subtalar joint disorders, injuries, and instability^11–14^, as well as for the design of implants and surgical procedures involving the subtalar joint^15–17^. Subtalar joint kinematics is also crucial for understanding the functional morphology and evolution of human bipedal walking, as the human talus and calcaneus are highly specialized in terms of morphology owing to adaptation to obligate bipedal locomotion^18–28^.

The human subtalar joint comprises three articular surfaces: the anterior, middle, and posterior facets. The anterior and middle facets of the calcaneus are continuously concave, articulating with the convex anterior and middle facets of the talar head and neck. In contrast, the posterior facet of the calcaneus is convex, articulating with the cylindrically concave posterior facet of the talus^29^. This alternating pattern of concave and convex surfaces, combined with ligamentous restraints, enables movement of the subtalar joint about an axis passing obliquely from anterior-medial-dorsal to posterior-lateral-plantar in the foot coordinate system, approximately through the neck of the talus, the tarsal sinus, and the lateral side of the calcaneus^30,31^. In humans, this axis is reportedly inclined at approximately 40 degrees from the horizontal plane in sagittal plane projection and roughly 20 degrees medially deviated from the midline or long axis of the foot in the horizontal plane^5,6,32–39^, although axis orientation varies considerably among individuals.

Assessments of human subtalar joint movement have often relied on the helical axis representation^37,40–43^. This method defines the spatial movement of a rigid body (e.g., the movement of the calcaneus from inversion to eversion) as a combination of rotation about an axis, known as the helical axis, and translation along the axis, like a screw^44,45^. However, the calcaneus may not be displaced along this rotational axis, which is directed anterodorsally in the sagittal plane. Recent kinematic analyses of the subtalar joint during human walking using biplane fluoroscopy demonstrated that translational movement of the calcaneus with respect to the talus occurs mainly in the mediolateral and anteroposterior directions, indicating that the motion is confined more to the horizontal plane rather than the sagittal plane^46,47^. Additionally, excessive medial translation of the calcaneus is a critical diagnostic marker in clinical evaluations of subtalar joint instability or laxity^48,49^. Subtalar mediolateral translation has been reported to be particularly pronounced in patients with ankle instability associated with morphological abnormalities of the ligaments during walking^50^. These clinical observations suggest that the innate translational mobility of the subtalar joint predominantly follows a mediolateral direction, consistent with the cylindrical geometry of the posterior facet whose anteromedial-to-posterolateral axis guides translation along its surface.

In the helical axis representation, translational motion occurs along the rotational axis, which includes only a slight mediolateral component. It is important to note, however, that a translational axis parallel to the rotational axis is not the only solution for defining the spatial movement of a rigid body by combining rotational and translational axes^51^. Mathematically, the two axes are not required to be parallel; they can have an arbitrary orientation relative to each other. In this proof-of-concept study, we propose a new approach for describing subtalar joint kinematics using a rotational axis that translates parallel to an anatomically defined axis of the subtalar joint—the cylindrical axis that approximates the posterior facet of the talus. This alternative kinematic representation offers a more physiologically intuitive and clinically useful description of subtalar joint motion, as it accurately reflects the joint’s anatomical constraints and functional behavior, making it well-suited for application in diagnosis, treatment planning, and surgical intervention. This study based on two cadaver specimens should be regarded as an exploratory pilot analysis serving as a technical demonstration rather than a generalizable model.

## Materials and methods

### Helical axis representation

**Fig. 1:**
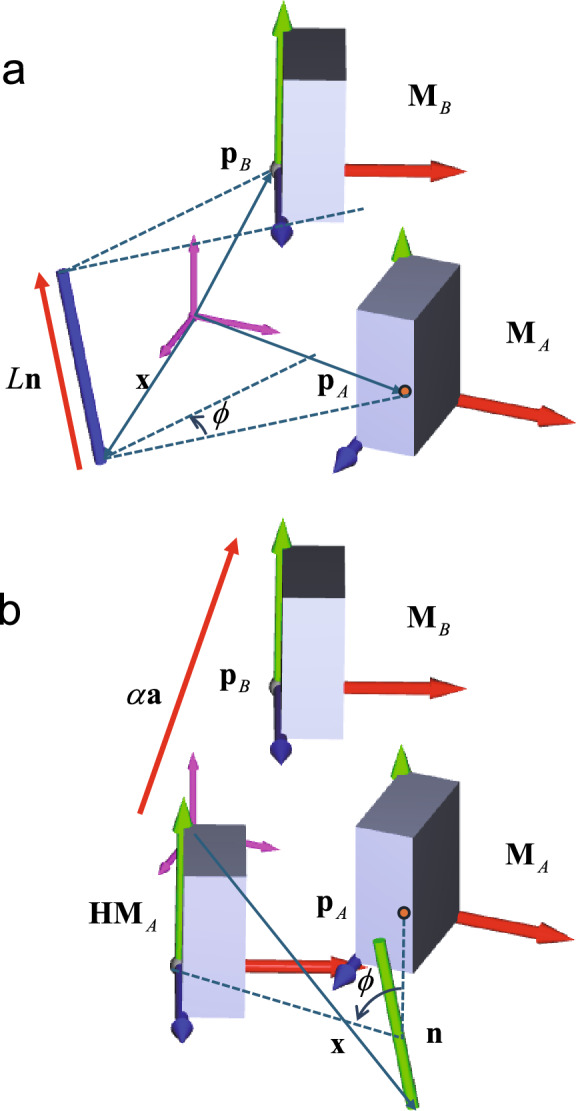
Comparison between the helical axis (**a**) and newly proposed (**b**) representations. Refer to the text for details on nomenclature. In the helical axis representation, the object (box) undergoes rotation with respect to **n** and translation along **n** by *L* (blue vector) in transformation from **M**_*A*_ to **M**_*B*_. In the newly proposed representation, the box undergoes rotation with respect to **n** (green vector) and translation along **a** by *α* (red vector) in transformation from **M**_*A*_ to **M**_*B*_. The angle $\phi$ remains constant in both the helical axis (**a**) and newly proposed (**b**) representations. However, their visual depiction may vary in the figure due to the different positions of the rotational axes.

A helical axis transformation describes the movement of a rigid body from one position to another using a rotational matrix **H** and a unit vector representing the helical axis **n**. When a given point on the rigid body before transformation, denoted as **p**_A_, is transformed to a new position, denoted as **p**_B_, via a helical axis transformation, the following equation can be derived (Fig. 1a)

1 $${\mathbf{H}}({\mathbf{p}}_{A} - {\mathbf{x}}) + L{\mathbf{n}} = {\mathbf{p}}_{B} - {\mathbf{x}}$$

where the positional vector **x** represents a point on the helical axis and *L* is the translation along the helical axis. The rotational matrix for a rotation about the helical axis **n** by an angle $\phi$ can be expressed using the Rodrigues’ formula^52^:

2 $${\mathbf{H}} = [(1 - \cos \phi ){\mathbf{n}} \cdot {\mathbf{n}}^{T} + (\cos \phi ){\mathbf{I}} + (\sin \phi ){\mathbf{S}}({\mathbf{n}})]$$

where **I** is the identity matrix and **S**(**n**) is the skew-symmetric matrix of **n**.

If the orientations of the rigid body (the orthonormal matrices of the body coordinate system) before and after the transformation, denoted as **M**_A_ and **M**_B_, respectively, are known, **H** can be calculated as

3 $${\mathbf{H}} = {\mathbf{M}}_{B} ({\mathbf{M}}_{A} )^{T}$$

The angle $\phi$ and the vector **n** can be calculated from **H** as^44,53^

4 $$\phi = \cos^{ - 1} [(tr({\mathbf{H}}) - 1)/2]$$

5 $${\mathbf{n}} = \frac{1}{2\sin \phi }\left[ {\begin{array}{*{20}c} {H_{32} - H_{23} } \\ {H_{13} - H_{31} } \\ {H_{21} - H_{12} } \\ \end{array} } \right]$$

where *H*_*ij*_ are elements of **H**. From Eqs. (1) and (2),

6 $$\begin{gathered} {\mathbf{p}}_{B} - {\mathbf{Hp}}_{A} = {\mathbf{t}} = ({\mathbf{I}} - {\mathbf{H}}){\mathbf{x}} + L{\mathbf{n}} \\ = \{ (1 - \cos \phi )({\mathbf{I}} - {\mathbf{nn}}^{T} ) - (\sin \phi ){\mathbf{S}}({\mathbf{n}})\} {\mathbf{x}} + L{\mathbf{n}} \\ \end{gathered}$$

If **p**_A_ and **p**_B_ are known, **x** can be calculated, but there are infinitely many solutions for **x** because all points on the helical axis should satisfy the equation. To find the unique solution for **x** such that **x** is perpendicular to the helical axis **n**, **x** is decomposed into two components: one that is perpendicular to **n** and another that is parallel to **n** as^53^

7 $${\mathbf{x}} = ({\mathbf{I}} - {\mathbf{nn}}^{T} ){\mathbf{x}} + {\mathbf{nn}}^{T} {\mathbf{x}}$$

Therefore, the first term ${\overline{\mathbf{x}}} = ({\mathbf{I}} - {\mathbf{nn}}^{T} ){\mathbf{x}}$ was substituted in Eq. (6) to obtain the unique solution ${\overline{\mathbf{x}}}$ as

8 $${\overline{\mathbf{x}}} = [(1 - \cos \phi ){\mathbf{I}} - (\sin \phi ){\mathbf{S}}({\mathbf{n}})]^{ - 1} ({\mathbf{t}} - L{\mathbf{n}})$$

Since ${\mathbf{n}}^{T} ({\mathbf{I}} - {\mathbf{H}}) = {\mathbf{0}}$, *L* can be calculated as

9 $$L = {\mathbf{n}}^{T} {\mathbf{t}}$$

In the helical axis representation, the position, direction, and length of the helical axis remain unchanged for both forward and inverse transformations of the rigid body.

### New representation by rotational axis translating along the talus posterior facet

In this study, the translation is assumed to occur along a unit vector **a**, in a direction that represents the axis of the cylinder that approximates the posterior facet of the talus. In this case, the transformation can be expressed by the following equation (Fig. 1b).

10 $${\mathbf{H}}({\mathbf{p}}_{A} - {\mathbf{x}}) + \alpha {\mathbf{a}} = {\mathbf{p}}_{B} - {\mathbf{x}}$$

where **a** is the unit vector unit representing the cylindrical axis, and *α* is the translation along the cylindrical axis. The calculation of $\phi$, **n** and **H** remains the same as previously stated. From Eqs. (10) and (2),

11 $$\begin{gathered} {\mathbf{p}}_{B} - {\mathbf{Hp}}_{A} = {\mathbf{t}} = ({\mathbf{I}} - {\mathbf{H}}){\mathbf{x}} + \alpha {\mathbf{a}} \\ = \{ (1 - \cos \phi )({\mathbf{I}} - {\mathbf{nn}}^{T} ) - (\sin \phi ){\mathbf{S}}({\mathbf{n}})\} {\mathbf{x}} + \alpha {\mathbf{a}} \\ \end{gathered}$$

As above, the unique solution ${\overline{\mathbf{x}}}$ and *α* can be calculated as

12 $${\overline{\mathbf{x}}} = [(1 - \cos \phi ){\mathbf{I}} - (\sin \phi ){\mathbf{S}}({\mathbf{n}})]^{ - 1} ({\mathbf{t}} - \alpha {\mathbf{a}})$$

13 $$\alpha = ({\mathbf{n}}^{T} {\mathbf{t}})/({\mathbf{n}}^{T} {\mathbf{a}})$$

In this proposed representation, while the direction of the rotational axes remains unchanged and identical to that of the helical axis, the positions of the rotational axes differ between forward and inverse transformations and from that of the helical axis.

### Specimens

For the comparative evaluation of subtalar joint kinematics using the conventional helical axis and the newly proposed transformations, the subtalar joint movements in response to tibial coronal inclination were investigated using cadaver specimens. Fresh-frozen cadaveric lower legs from two human donors (Foot A and Foot B; both female, aged 90 and 57 years at the time of death) were obtained for CT measurements. The specimens were donated to Keio University School of Medicine with the informed consent of the families of all the donors and confirmed to be free of foot and ankle pathologies by visual and radiographic inspection. The cadaver foot study was approved by the ethics committee of the School of Medicine, Keio University (20150385), and by the Office for Life Science Research Ethics and Safety, The University of Tokyo (19–296, 23–563). All methods were performed in accordance with the relevant guidelines and regulations.

### Quantifying subtalar joint motion through CT scan analysis

**Fig. 2:**
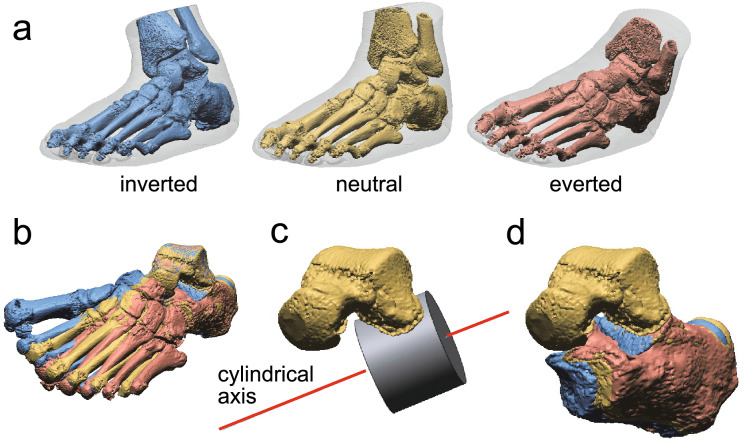
Workflow from CT scanning and 3D reconstruction to axis calculation and comparison. (**a**) CT scanning in neutral, inverted, and everted foot postures followed by 3D reconstruction. (**b**) Talar surface registration using the iterative closest point algorithm. (**c**) Calculation of the cylindrical axis by fitting a best-fit surface to the posterior facet of the talus. (**d**) Calculation of the rotation and translation of the calcaneus relative to the talus based on both the conventional helical axis representation and the proposed representation with a rotational axis translating along the cylindrical axis of the talus posterior facet.

CT scan data of the lower legs were acquired using Aquilion ONE (Canon Medical Systems, Otawara, Japan) at Keio University Hospital. Each foot was placed at plantigrade on the bottom panel of the expanded polystyrene box (inside dimension, 420 × 220 × 200 mm) placed on the CT table. The long axis of the foot, defined by the line connecting the calcaneal tuberosity and the head of second metatarsal, was approximately aligned with the anteroposterior axis of the CT table. The CT scans were performed in three foot postures (Fig. 2a): (1) the neutral posture with the tibial inclination angle of approximately 0 degrees in the both coronal and sagittal planes (the plantar surface of the foot was in approximately 90 degrees relative to the tibia in the both planes); (2) the inverted foot posture with approximately 20–25 degrees of the medial tibial inclination; and (3) the everted foot posture with approximately 20–25 degrees of lateral tibial inclination. The pixel size of the CT images ranged from 0.27 to 0.37 mm, and the slice interval was 0.5 mm.

From these scans, the hindfoot comprising the talus and calcaneus was extracted by a thresholding technique and three-dimensionally reconstructed using a marching cube algorithm, both with the image analysis software Mimics 24.0 (Materialise, Leuven, Belgium) (Fig. 2a). Then, the reference coordinate system of the talus in the neutral posture was defined. Recently, the principal axes have been used as the reference coordinate system of the talus^40,54^. However, traditionally, the orientation of the joint axis of the subtalar joint has been represented with respect to the plantar surface and longitudinal axis of the foot^30,33^, which better approximate the coordinate system used in kinematic investigations. Therefore, in the present study, we defined the talus local coordinate system in such a way that the x -axis is parallel to the long axis of the foot in the neutral posture, the y-axis perpendicular to the x-axis and parallel to the plantar surface of the foot and the z-axis is the cross product of the two vectors. Subsequently, the hindfeet (talus and calcaneus) in inverted and everted positions were aligned with the hindfoot in the neutral posture in two procedural steps. First the inverted and everted feet were registered to the neutral talus (Fig. 2b) based on the surface morphology of the talus utilizing the iterative closest point (ICP) algorithm by a commercial software Geomagic XOS64 (3D Systems, Rock Hill, South Carolina, USA). Such a registration allows visualization and quantification of the calcaneus movement with respect to the talus. Next, the calcaneus in the neutral posture is registered and aligned with its counterparts in the inverted and everted postures again utilizing the ICP algorithm. This registration process allows the exact calculation of translation and rotation necessary to reconcile the three calcaneal postures. Subsequently, cylindrical and helical axes are determined based on these calculations, as outlined previously, assuming rigid body motion of the bones and a linear cylindrical approximation of the posterior facet of the talus (Fig. 2c,d). The cylindrical axis was defined by calculating best-fit surface to the posterior facet of the talus. No continuous interpolation of motion was performed; instead, rigid body transformations between these static positions were used as the basis for calculating the rotational and translational axes.

## Results

**Fig. 3:**
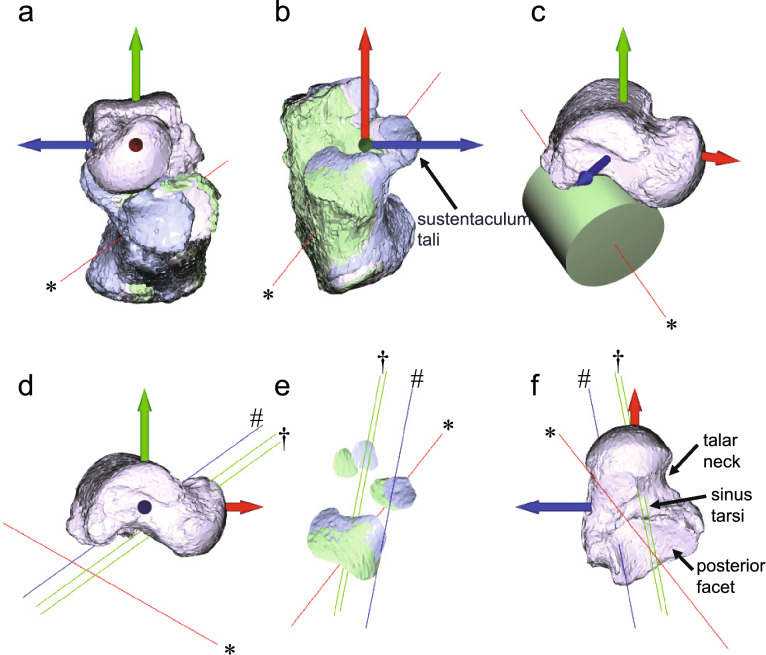
Movement of the Foot A subtalar joint. (**a**) Frontal and (**b**) superior views illustrating the motion of the calcaneus relative to the talus and the cylindrical axis (red, *). (**c**) Approximation of the posterior facet of the talus using a cylindrical model. (**d**) Medial (**e**) superior and (**f**) inferior views showing the helical axis (blue, #) and newly proposed rotational axis (green, †) translating along with the cylindrical axis (red, *).

**Fig. 4:**
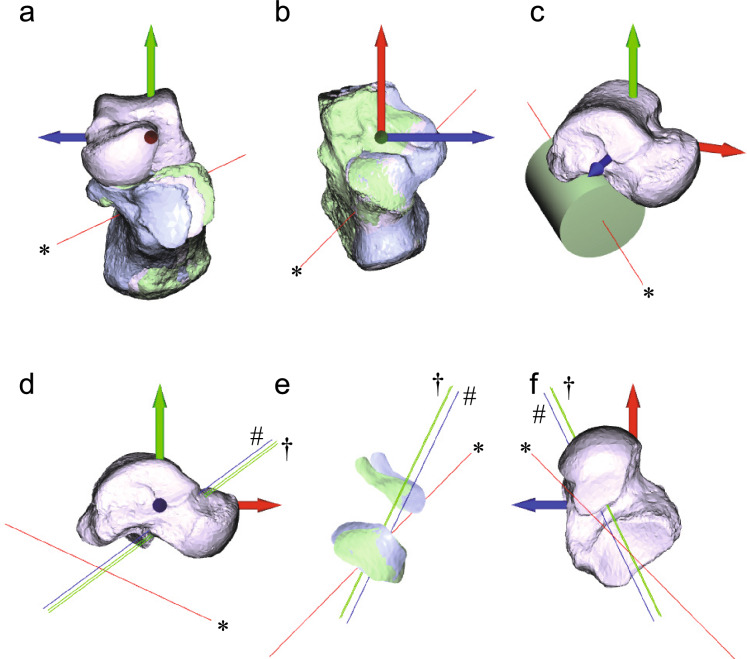
Movement of the Foot B subtalar joint. Refer to the legend of Fig. 3 for details.

Figures 3a,b and 4a,b illustrate the movement of the calcaneus relative to the talus in neutral, inverted, and everted foot postures. In the current study, the root mean square of the ICP residuals was at most 0.042 mm, indicating a high degree of alignment accuracy, and the final alignment was visually confirmed. The cylindrical approximation of the posterior facet of the talus (Figs. 3c and 4c) is shown in red in each figure. Figures 3d,e,f and 4d,e,f display the respective helical axis (blue line) and the newly proposed rotational axis representation translating along the cylindrical axis (green lines). Notably, there are two rotational axes in the proposed representation because their positions differ between forward and inverse transformations. However, these axes remain parallel and are situated close to each other. The rotational magnitudes (17.6° and 19.3° for Foot A and Foot B, respectively; Table 1) and the directions of the rotational axes are consistent between the helical axis (blue line) and the proposed rotational axes (green lines). However, their spatial locations differ. The helical axis is positioned more posterosuperomedially compared to the proposed rotational axes (Figs. 3d,e,f and 4d,e,f). The proposed rotational axes pass above the anterior talocalcaneal facet, traverse the tarsal canal and the junction of the tarsal sinus, and extend beneath the posterior talocalcaneal facet (Figs. 3e,f and 4e,f).

**Table 1:** Rotational and translational movements of the subtalar joint based on the new representation (rotational axis translating along the posterior facet of the talus) and the conventional helical axis representation.

Foot	Rotation [°]	Translation along talus posterior facet [mm]	Translation along helical axis [mm]
A	17.6	4.0	1.7
B	19.3	1.3	0.7

In the transformation of the calcaneus from an everted to an inverted posture using the helical axis, the calcaneus undergoes a superoanteromedial translation along the helical axis, followed by rotation. However, in the proposed method, the calcaneus is translated inferoanteromedially along the cylindrical axis before rotation, which more closely aligns with the observed movements of the calcaneus (Figs. 2a,d and 3a,d). The translation magnitude along the helical axis was calculated to be 1.7 mm for Foot A and 0.7 mm for Foot B (Table 1). In contrast, with the proposed method, the translation magnitudes along the cylindrical axis were 4.1 mm and 1.3 mm, respectively, indicating that the proposed method captures a significantly larger translation (Table 1).

## Discussion

In the helical axis representation, translational motion occurs along the rotational axis. This representation has recently been applied in humans to estimate subtalar axis orientation and position based on rigid body displacements measured from CT or MRI^40,41^. The helical axis representation in our examples appears similar to those reported in these studies, with the rotational axis positioned more posteromedially than in our newly proposed representation. However, observing actual calcaneus movement relative to the talus reveals a translation that aligns more closely with the axis of the cylinder approximating the posterior facet of the subtalar joint of the talus (red line) than with the helical axis (blue line) (Figs. 3a,b,e and 4a,b,e). When assuming translation along the cylindrical axis, the calculated rotational axis is located more anteroinferolaterally than the helical axis, which is typically described as passing opposite the middle facet of the subtalar joint near the origin and insertion of the interosseous talocalcaneal ligament in the tarsal canal^41,55,56^. However, our alternative estimation of the rotational axis location may be more accurate, as the rotational pivot of the subtalar joint is likely positioned more anterolaterally due to the cervical ligament^10,31^ and the anterior capsular ligament^55^, which are located lateral to the interosseous talocalcaneal ligament. These three ligaments work together to tightly connect and stabilize the talus and calcaneus^10,56,57^, effectively serving as the rotational pivot of the subtalar joint.

The present study proposes a novel, alternative approach for characterizing subtalar joint kinematics by having the rotational axis translate parallel to the cylindrical axis approximating the posterior facet of the subtalar joint of the talus. This innovative kinematic representation may provide a more intuitive and clinically relevant depiction of subtalar joint motion. In the future, it could potentially contribute to the diagnosis and evaluation of subtalar joint instability, as excessive medial translation is a critical marker in clinical settings. The greater translation values observed under the cylindrical model (Table 1) may have clinical implications, such as influencing surgical reference planes for talocalcaneal fusion or the alignment of subtalar implants. Although direct pathological thresholds were not examined, the magnitude of mediolateral translation in our model approaches values reported in cases of subtalar instability, suggesting that a translation axis aligned with the cylindrical axis may better capture clinically relevant motion patterns. While these interpretations remain speculative and require validation in clinical studies involving patients with subtalar pathology, this methodology, by refining the ability to characterize complex subtalar movements, could potentially improve the understanding of pathological subtalar joint mechanics and inform targeted therapeutic interventions. Furthermore, the proposed representation may also have potential relevance for surgical planning, prosthesis alignment, and rehabilitation strategies aimed at improving mediolateral stability. These possibilities warrant further investigation in future clinical studies. It must be noted, however, that the clinical application of the present method would require acquisition of detailed talar morphology, which can be obtained from CT imaging. While this requirement may limit immediate use in routine clinical practice, the method could be integrated into current workflows in settings where CT-based 3D modeling is available. Advances in biplanar fluoroscopy^46,47^ or upright CT scanner^58,59^, or other imaging modalities capable of capturing talar morphology, may further enhance its clinical feasibility.

Compared with multi-axis approaches such as Euler angles, the helical axis representation has the advantage of describing the motion between two rigid body positions with a single axis and a rotation/translation pair, avoiding the difficulty of intuitively understanding how rotation occurs in three-dimensional space from separate component angles which depends on the chosen rotation sequence. The cylindrical-axis method proposed in this study retains these advantages of the helical axis while incorporating an anatomically defined translation direction based on the posterior facet of the talus.

However, it is important to note that mathematically, the axis of translation could occur in any direction, and the choice of axis (or direction) alters the location of the rotational axis. Although we argue that selecting the cylindrical axis for translation in the subtalar joint results in a reasonable representation of subtalar joint movement, as observed in the present study, this finding is based on static, non-weight-bearing CT scans, whereas our motivation stems from in vivo biplanar fluoroscopic data. While the proposed representation could, in principle, be transferred to dynamic functional tasks, its validity in such contexts remains untested and should be confirmed in future studies. Another limitation of this study is that only three static foot positions (neutral, inverted, and everted) were analyzed, which may not fully capture the continuous nature of subtalar joint motion. Future studies using dynamic or more densely sampled postures could provide a more comprehensive characterization of the joint’s kinematics. In addition, the reproducibility of the cylindrical axis definition across specimens, and its sensitivity to segmentation, was not directly evaluated in the present study. While we expect the procedure to be generally robust owing to the clear anatomical boundaries of the posterior facet, future studies should assess inter- and intra-operator variability and test the method across a larger sample. Lastly, as this is a cadaveric study, muscle forces were absent, which may affect joint kinematics compared to in vivo conditions. However, the subtalar joint motion is primarily determined by the passive morphological and mechanical properties of the joint, namely, bone morphology and ligamentous structures. Although the present study quantified this inherent passive motion as determined by the subtalar joint structure, subtalar joint kinematics during weight-bearing or dynamic activities such as walking or running in vivo should be examined in future studies using the proposed method.

## Data Availability

The datasets used and/or analyzed during the current study, except for the CT scan images, are available from the corresponding author upon reasonable request. The CT scan data used in this study are subject to restrictions due to participant privacy concerns and are restricted by the institutional review board.
